# Effects of *Sapindus mukorossi* Seed Oil on Proliferation, Osteogenetic/Odontogenetic Differentiation and Matrix Vesicle Secretion of Human Dental Pulp Mesenchymal Stem Cells

**DOI:** 10.3390/ma13184063

**Published:** 2020-09-13

**Authors:** Shiau-Ting Shiu, Wei-Zhen Lew, Sheng-Yang Lee, Sheng-Wei Feng, Haw-Ming Huang

**Affiliations:** 1 School of Dentistry, College of Oral Medicine, Taipei Medical University, Taipei 11031, Taiwan; aaa.50628@hotmail.com (S.-T.S.); b202094090@tmu.edu.tw (W.-Z.L.); seanlee@tmu.edu.tw (S.-Y.L.); 2 Department of Dentistry, Shuang Ho Hospital, Taipei Medical University, New Taipei City 23561, Taiwan; 3 Department of Dentistry, Wan-Fang Medical Center, Taipei Medical University, Taipei 11031, Taiwan; 4 Division of Prosthodontics, Department of Dentistry, Taipei Medical University Hospital, Taipei 11031, Taiwan; 5 Graduate Institute of Biomedical Optomechatronics, College of Biomedical Engineering, Taipei Medical University, Taipei 11031, Taiwan

**Keywords:** *Sapindus mukorossi*, dental pulp mesenchymal stem cell, proliferation, osteognesis, odontogenesis, matrix vesicle

## Abstract

Stem cells have attracted great interest in the development of tissue engineering. However, the self-regeneration and multi-differentiation capabilities of stem cells are easily impaired during cell transplantation. Recent studies have demonstrated that *Sapindus mukorossi* (*S. mukorossi*) seed oil has various positive biological effects. However, it is not yet clear whether *S. mukorossi* seed oil can increase the growth and differentiation of dental pulp mesenchymal stem cells (DPSCs). The aim of this study is to investigate the effects of *S. mukorossi* seed oil on the proliferation and differentiation of DPSCs. DPSCs with and without *S. mukorossi* seed oil, respectively, were evaluated and compared. The viabilities of the cells were assessed by MTT tests. The osteogenetic and odontogenetic capacities of the DPSCs were tested using Alizarin red S staining and alkaline phosphatase (ALP) activity assays. In addition, real-time PCR was performed to examine the gene expression of ALP, BMP-2 and DMP-1. Finally, extracellular matrix vesicle secretion was detected via scanning electron microscopy. No significant difference was observed in the viabilities of the DPSCs with and without *S. mukorossi* seed oil, respectively. However, under osteogenic and odontogenic induction, *S. mukorossi* seed oil increased the secretion of mineralized nodules and the ALP activity of the DPSCs (*p* < 0.05). The ALP gene expression of the differentiation-induced DPSCs was also enhanced. Finally, a greater secretion of extracellular matrix vesicles was detected in the DPSCs following odontogenic induction complemented with *S. mukorossi* seed oil. Overall, the present results show that *S. mukorossi* seed oil promotes the osteogenic/odontogenic differentiation and matrix vesicle secretion of DPSCs.

## 1. Introduction

Tissue engineering involves the application of biological and engineering principles to the development of bio-replacement cells or materials for the repair, maintenance, or functional enhancement of human tissue [1]. In effecting tissue repair, the cells or materials are transplanted directly to the injury site and eventually become part of the patient’s body. However, to avoid complications, the biological properties of the transplanted cells must be properly understood in order to ensure their successful integration with the host tissue [2]. 

Adult stem cells have been successfully isolated from various human tissues, including bone marrow, adipose tissue, dental pulp, muscle, and placenta [3,4,5]. Moreover, recent studies have shown that the plasticity of stem cell differentiation means that adult stem cells are not restricted only to differentiation in the same germ layer [6]. Consequently, stem cell-based tissue engineering has attracted great interest within the medical and scientific communities in recent decades. Dental pulp mesenchymal stem cells (DPSCs) are multipotent stromal cells with a strong ability for self-renewal and multi-lineage differentiation [7,8]. Compared with other stem cells, the advantages of using DPSCs are the cells can be obtained from discarded teeth in a non-invasive manner with minimal risk of complications [9,10]. Furthermore, DPSCs have many favorable properties for tissue engineering and regenerative medicine, such as a high proliferation rate, a good odontogenic/osteogenic differentiation potential, and excellent immunomodulatory properties [7,11]. However, DPSCs also have several important limitations for therapeutic use, including a low harvesting quantity and a degradation of properties following long-term culturing [7,11]. Extrinsic factors such as the health status of the donor, aging, the inflammatory environment and a low level of oxygen also have a significant effect on the efficiency of clinical cell transplantation [11,12]. Many approaches for addressing these limitations have been proposed, including pre-conditioning the cells with specific molecules, bioactive compounds, natural plant extracts, physical stimulation, and three-dimensional (3D) cell culturing [13,14,15,16,17].

**Figure 1:**
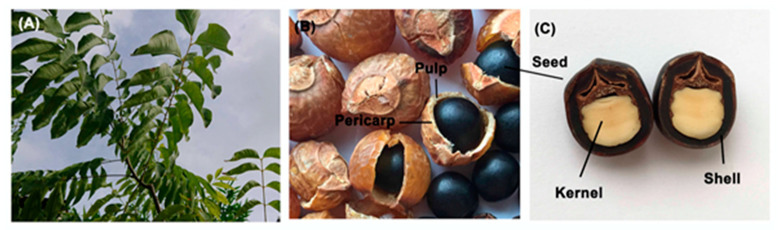
(**A**) Fresh *S. mukorossi* fruit and leaf. (**B**) After maturation, the *S. mukorossi* fruit turn dark brown and contain a black seed. (**C**) The seed consists of an oil-enriched pulp covered with a hard shell.

There is mounting evidence to show that natural plant extracts possess the ability to promote the proliferation or differentiation of various mesenchymal stem cells (MSCs) [14,18,19,20]. For example, the components of Safflower seed oil and palmitic acid can differentiate embryonic neural stem cells into neurons [21]. Similarly, the Chinese herb *Fallopia multiflora* (He Shou Wu in Chinese) prompts the self-renewal of human DPSCs via the AMPK/ERK/SIRT1 axis [22]. *Sapindus mukorossi* (*S. mukorossi*), listed in the Chinese Compendium of Materia Medica (Bencao Gangmu in Mandarin) some 500 years ago with the name of Wu Huan Zi, is also regarded as a valuable medicinal plant (Figure 1A). It is widely distributed from Japan to tropical India [23,24] and is characterized by an abundance of saponins within its pericarp and pulp (Figure 1B), which render it an excellent washing agent for the body, hair and clothes [25,26]. *S. mukorossi* seed is also useful for treating a number of medical disorders, such as vomiting, excessive salivation, epilepsy, eczema and psoriasis [24,27]. *S. mukorossi* extracts have many unique biological and pharmacological effects, including anti-inflammatory, antimicrobial, antifungal, free radical scavenging, antioxidant and anti-tumor activities [19,28,29,30]. In addition to saponins, the kernel of *S. mukorossi* seed contains around 30% oil (Figure 1C). In vitro studies have shown that this oil not only promotes cell proliferation and migration, but also has significant anti-inflammatory effects. Furthermore, in vivo animal tests have shown that *S. mukorossi* seed oil has strongly positive effect on the skin wound healing [19].

*S. mukorossi* seed oil was reported as a plant oil riches in phytosterols and arachidonic acid (23.85%), which increases its anti-inflammatory effects and promotes wound healing [31]. However, the effects of *S. mukorossi* seed oil on the biological response of MSCs are still unclear [14]. Accordingly, the present study evaluates the effectiveness of *S. mukorossi* seed oil on the proliferation, differentiation and matrix vesicle secretion of DPSCs in in vitro experiments. 

## 2. Materials and Methods

### 2.1. DPSCs Isolation and Culture

All the experimental procedures were approved by the Joint Institutional Review Board of Taipei Medical University (TMU-JIRB No. 201503064). Prior to teeth extraction, formal written consent was received from each donor following a full explanation of the experimental procedures and purpose. DPSCs were isolated from the freshly extracted teeth using the method described in previous studies [7,8]. Briefly, healthy premolars or molars were obtained from the donors as the natural outcome of orthodontic treatment. The pulp tissue was carefully removed from each tooth by a sterile mortar and pestle. The tissue was washed with PBS for three times. Then the tissue was minced into pieces, and then cultured in 3.5 cm diameter Petri dishes at 37 °C in a 5% CO_2_ environment. Culturing was performed using alpha minimum essential medium (α-MEM, Gibco/Invitrogen, Carlsbad, CA, USA), for which each liter of medium comprised 10.2 g of powder-packed α-MEM, 2.2 g of sodium bicarbonate (Sigma-Aldrich, St. Louis, MO, USA), 10 mL of antibiotic-antimycotic solution (Sigma-Aldrich), 5 mL of L-ascorbic acid 2-phosphate, and 150 mL of fetal bovine serum (FBS, Gibco/Invitrogen). When the cell cultures reached ≥80% confluence, the pulp cells were detached and passaged with 0.5% trypsin-EDTA solution. After using a 70-µm strainer (BD Falcon, San Jose, CA, USA) to isolated DPSCs, the cells were then cultured in 10-cm diameter Petri dishes for future investigation.

### 2.2. S. mukorossi Seed Oil Preparation

*S. mukorossi* seed oil was prepared using the method described in a previous study by the present group [19]. Briefly, the kernels were separated from the plant seed and the oil was extracted by a cold press method. The extracted oil was filtered through a 0.45 µm pore size filter and stored in a 4 °C environment for subsequent experimental use. The seed oil had a hydrophobic characteristic. Thus, prior to the culture tests, the oil was mixed with culture medium using dimethyl sulfoxide (DMSO, Sigma-Aldrich, St. Louis, MO, USA) as an emulsifier with a DMSO to seed oil mixing ratio of 5:2 (*v*/*v*).

### 2.3. Cell Viability Assay 

**Figure 2:**
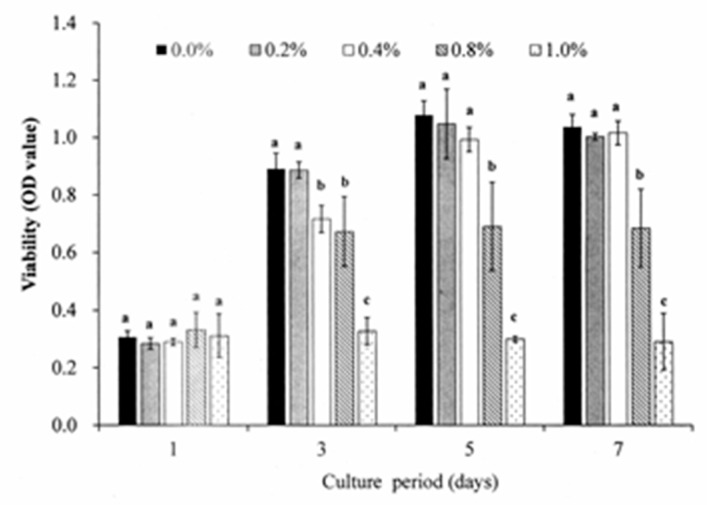
Viabilities of DPSCs cultured with various DMSO concentrations for a maximum period of 7 days. A DMSO concentration of 0.2% has no significant effect on the DPSC viability irrespective of the incubation period. Note that OD values not sharing the same letter are significantly different (*p* < 0.05).

**Figure 3:**
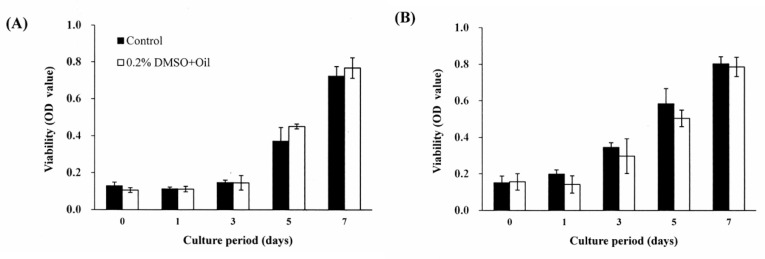
Effects of *S. mukorossi* seed oil on viability of DPSCs seeded with cell densities of: (**A**) 2 × 10^4^ cells/mL and (**B**) 5 × 10^4^ cells/mL. For both seeding densities, similar tendencies are noted when comparing the experimental group and control group. In particular, no significant difference in the cell viabilities of the two groups is observed over the entire 7-day incubation period.

The effect of DMSO on the DPSC viability was examined by means of MTT assays performed using *S. mukorossi seed oil*/medium solutions with DMSO concentrations of 0.2–1.0% (*v*/*v*) and an incubation period of up to seven days. Based on the test results, a stock solution of *S. mukorossi seed oil*/medium with a DMSO concentration of 0.2% was prepared for the following experiments (Figure 2). The effect of *S. mukorossi* seed oil on the viability of the DPSCs was assessed by MTT assays using DPSCs cultured with medium only as a blank control group. For the experimental group, DPSCs with densities of 2 × 10^4^ cells/mL and 5 × 10^4^ cells/mL were cultured with medium contained *S. mukorossi* seed oil. After 1, 3, 5 and 7 days of culture, 3-(4,5-dimenthylthiazol-2-yl)-2,5-diphenyltetrasoliumbromide (MTT, Roche Applied Science, Mannheim, Germany) solution with a concentration of 5 mg/mL was then added to the culture dishes for 4 h. The optical densities of the formazan crystals dissolved with the DMSO were detected at a wavelength of 570 nm and a reference wavelength of 690 nm using a microplate reader (EZ Read 2000, Biochrom Ltd., Cambridge, UK). According to the cell viability test results (Figure 3), DPSCs with an initial seeding density of 2 × 10^4^ cells/mL were adopted for all of the following differentiation experiments.

### 2.4. Effect of S. mukorossi Seed Oil on Osteogenesis and Odontogenesis Differentiation of DPSCs

DPSCs were seeded in a 24-well culture dish with a density of 2 × 10^4^ cells/mL. After the DPSCs reached 80% confluence, the culture medium was replaced with osteogenesis induction medium (OTM) or odontogenesis induction medium (ODM). The OTM medium comprised α-MEM (Gibco/Invitrogen), 90 mM KH_2_PO_4_ (J.T. Baker, Phillipsburg, NJ, USA) and 0.1 μM Dexamethasone (Sigma-Aldrich). Meanwhile, the ODM medium consisted of α-MEM with 10 nM Dexamethasone, 50 μg/mL ascorbic acid, and 5 mM β-glycerophosphate (Sigma-Aldrich). The OTM or ODM was replaced every 2 to 3 days. The effects of *S. mukorossi* seed oil on the osteogenesis and odontogenesis differentiation of the DPSCs were evaluated by performing co-culturing tests using *S. mukorossi* seed oil and OTM (OTM-oil) or ODM (ODM-oil) as the experimental groups and osteogenesis induction-free medium (OTFM) or odontogenesis induction-free medium (ODFM) as the blank control groups. After 14 days of induction, the DPSCs in the various groups were fixed with 4% paraformaldehyde and stained with 2% Alizarin red S staining solution (Alizarin red S, Sigma-Aldrich). Briefly, 250 µL of staining solution was added to each well for 2 min. After removing the staining solution, the DPSCs were washed with PBS. An illumination microscope (Eclipse TS100, Nikon Corporation, Tokyo, Japan) connected with a digital camera (SPOT Idea, Diagnostic Instruments, Inc., Sterling Heights, MI, USA) was then used to observe the stained cells. Following the observation process, the cells were treated with 10% acetic acid and 10% ammonium hydroxide for qualification using a spectrophotometer at a wavelength of 405 nm.

### 2.5. Alkaline Phosphatase Activity Assay 

The alkaline phosphatase (ALP) activities of the DPSCs in the ODFM, ODM and ODM-oil groups were determined. The procedure was determined by testing the rate of conversion of p-nitrophenyl phosphate to p-nitrophenol at a pH of 10.2. Briefly, DPSCs were seeded in a 6-well culture dish with a density of 2 × 10^4^ cells/mL and were maintained in an incubator for 24 h. The culture medium was then replaced with odontogensis differentiation induction medium with or without *S. mukorossi* seed oil, as described above. After 5, 10 and 15 days of induction, the media were removed and the remaining cells were washed three times with PBS. 300 μL CelLytic^TM^ cell lysis reagent (Sigma-Aldrich) was then added to each sample to isolate the intracellular protein. After pipetting to break the cells, 200 µL Bio-Rad protein assay reagent (BCA Protein Assay Kit; Pierce, Rockford, IL, USA) was added to 10 μL of cell lysate and incubated for 10 min. The absorbance of each sample was then read spectrophotometrically at a wavelength of 590 nm to determine the total protein content. Finally, 50 μL of each cell lysate was mixed with 250 μL of ALP assay reagent in a 96-well microtiter plate and the optical absorbance was measured at 405 nm. 

### 2.6. Quantitative Real-Time Polymerase Chain Reaction Analysis

Quantitative real-time polymerase chain reaction (qRT-PCR) tests were performed to determine the osteognenesis- and odontogenesis-related gene expressions of the DPSCs activated by the *S. mukorossi* seed oil. Briefly, total ribonucleic acid (RNA) was obtained using a Novel Total RNA Mini Kit (NovelGene Biotech, Taipei, Taiwan) according to the instruction of the manufacturer. The harvested RNA was used to synthesize complementary deoxyribose nucleic acid (cDNA). A High-Capacity cDNA Reverse Transcription Kit (Applied Biosystems™, Foster City, CA, USA) was carried out to complete this procedure. By adding with FastStart Universal SYBR Green Master dye (Roche Applied Science, Mannheim, Germany), the target cDNA was amplified using a real-time DNA thermal analyzer (Rotor-gene 6000; Corbett Life Science, Sydney, Australia). The expressions of ALP, bone morphogenetic protein-2 (BMP-2) and dentin matrix acidic phosphoprotein 1 (DMP-1) were evaluated (see Table 1). To normalize the fluorescence signals, the human GAPDH gene was analyzed synchronously to be an endogenous control. In addition, the comparative ΔC_T_ method was used to determine the relative amount of each target sequence. Finally, the relative expression levels of the different genes were normalized using the 2^−ΔΔCT^ method as descripted previously [7,8].

### 2.7. Scanning Electron Microscopy Assay

The morphologies of the DPSCs after odontogenesis induction with and without *S. mukorossi* seed oil were observed using scanning electron microscopy (SEM, Hitachi SU-3500, Hitachi High Technologies, Minato-ku, Tokyo, Japan). Prior to the observation process, the DPSCs were cultured on 1-cm round glass coverslips with ODFM, ODM and ODM-oil respectively. After 14 days of incubation, the DPSCs were fixed with glutaraldehyde, washed with PBS and dehydrated with a graded series of ethanol. The slides were then taken out for critical point drying and sputter-coated with Au.

### 2.8. Statistical Analysis

The experimental data were all presented as mean ± standard deviation (SD). The statistical analyses were performed using commercialized statistic software (SPSS, Inc., Chicago, IL, USA). The differences between tested groups were evaluated using the Student’s *t*-test. In addition, one-way analysis of variance (ANOVA) tests followed by Tukey’s post hoc test were performed for multiple comparisons. In this study, a *p* value lower than 0.05 was considered to be statistically significant.

## 3. Results

### 3.1. Cell Viability Assays

Figure 2 shows the cell viability analysis results for the DPSCs cultured with various concentrations of DMSO. For the cells cultured with DMSO-free medium, the viability increases steadily for the first five days and reached a plateau after seven days. When 0.2% DMSO is added to the culture medium, no significant difference is observed in the cell viability over the entire experimental period. However, when the DMSO concentration is increased to 0.4%, a significant reduction in the cell viability occurs after three days. For DMSO concentrations greater than 0.4%, the cell viability reduces significantly (*p* < 0.05) over the seven-day period. In general, the results show that DMSO has no toxicity effect on the DPSCs provided that it is present only in very low concentrations (i.e., <0.2%). Figure 3A,B show the effects of *S. mukorossi* seed oil addition on the DPSC viability for initial seeding densities of 1 × 10^4^ cells/mL and 5 × 10^4^ cells/mL, respectively. For both seeding densities, the addition of *S. mukorossi* seed oil to the DMSO/medium solution has no significant effect on the viability of the DPSC cells.

### 3.2. Alizarin Red S Staining and Osteogenesis Quantification

**Figure 4:**
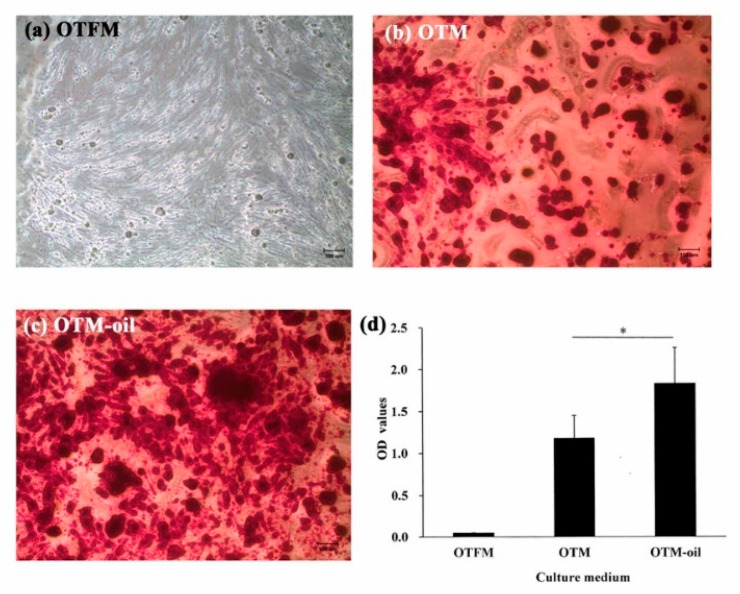
Osteogenic differentiation capacities of DPSCs as assessed by Alizarin red S staining after 14-day culture period. (**a**) No mineralized nodule deposition is observed when the DPSCs are cultured in general medium. (**b**) Following osteogenic medium induction, observable mineralized nodule deposition occurs. (**c**) Following co-culturing with osteogenic medium and *S. mukorossi* seed oil, the DPSCs secrete more abundant mineralized nodules. (**d**) Quantification of the Alizarin red S staining results shows a significant difference between the OTM and OTM-oil groups (* *p* < 0.05). (Note OTFM: osteogenesis induction-free medium; OTM: osteogenesis induction medium; and OTM-oil: osteogenesis induction medium with *S. mukorossi* seed oil.). Scale bars for **a**, **b** and **c** denote 100 µm.

As shown in Figure 4a, the DPSCs cultured with OTFM showed only very minor staining and mineralized nodule deposition. However, for the DPSCs cultured with OTM, obvious dark-red stains corresponding to calcium ion deposits were observed (Figure 4b). Furthermore, for the DPSCs co-cultured with *S. mukorossi* seed oil (OTM-oil), strong positive staining was observed throughout the entire cellular construct (Figure 4c). Figure 4d presents a quantitative analysis of the Alizarin Red S staining results The Ca contents of the OTFM, OTM and OTM-oil groups are seen to be 0.048 ± 0.004, 1.177 ± 0.269 and 1.882 ± 0.428, respectively. A significant difference exists between the OTM and OTM-oil groups (*p* < 0.05). In other words, the addition of *S. mukorossi* seed oil significantly improves the osteogenic differentiation degree of the DPSCs. 

### 3.3. Alizarin Red S Staining and Odontogenesis Quantification

**Figure 5:**
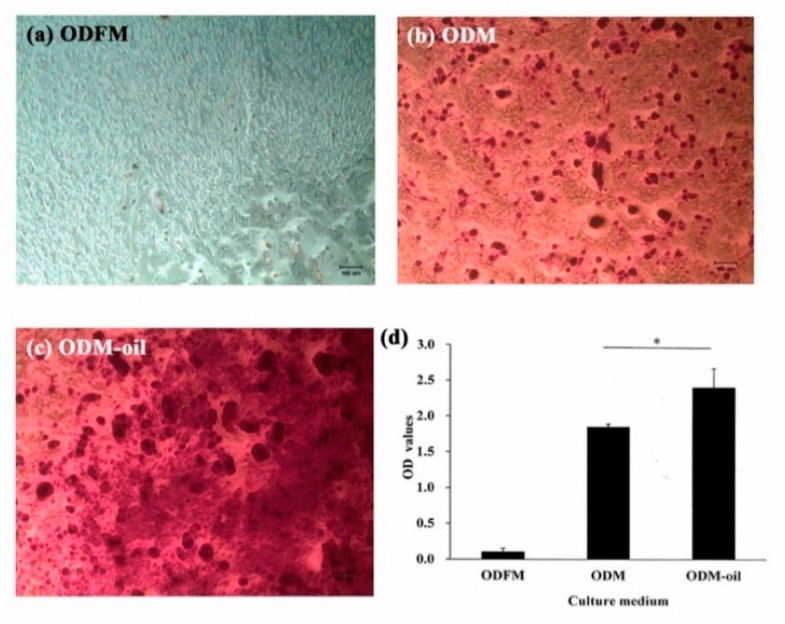
Odontogenic differentiation capacities of DPSCs as assessed by Alizarin red S staining after 14-day culture period. (**a**) No mineralized nodule deposition is observed when the DPSCs are cultured in general medium. (**b**) Following odontogenic medium induction, observable mineralized nodule deposition occurs. (**c**) Following co-culturing with odontogenic medium and *S. mukorossi* seed oil, the DPSCs secrete more abundant mineralized nodules. (**d**) Quantification of the Alizarin red S staining results shows a significant difference between the ODM and ODM-oil groups (* *p* < 0.05). (Note ODFM: odontogenesis induction-free medium; ODM: odontogenesis induction medium; and ODM-oil: odontogenesis induction medium with *S. mukorossi* seed oil.). Scale bar for **a**, **b** and **c** denote 100 µm.

As shown in Figure 5, the DPSCs exhibited obvious differences in the mineralization extent when cultured with different ODM media. In particular, the DPSCs cultured with ODFM showed very little positive staining (Figure 5a). However, significant calcium deposits were observed in the ODM group (Figure 5b). Furthermore, the extent of the calcium deposition increased when the DPSCs were co-cultured with ODM and *S. mukorossi* seed oil (ODM-oil) (Figure 5c). The quantitative analysis results presented in Figure 5d show that the ODFM, ODM and ODM-oil groups have average Ca contents of 0.102 ± 0.053, 1.847 ± 0.042 and 2.396 ± 0.268, respectively. The Ca contents of the ODM and ODM-oil groups are significantly different (*p* < 0.05). In other words, the addition of *S. mukorossi* seed oil also has a positive effect on the odontogenic differentiation capacity of the DPSCs.

### 3.4. Alkaline Phosphatase Activity Assay

**Figure 6:**
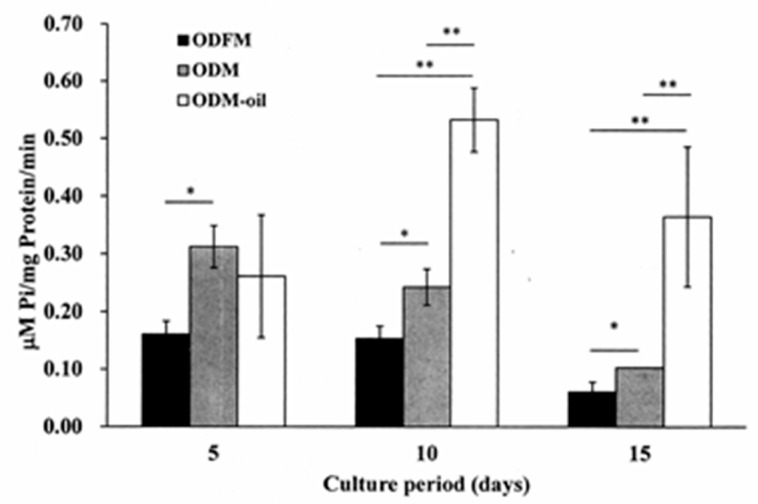
Alkaline phosphatase activity assessment of three experimental groups following culture periods of 5, 10 and 15 days. After culture periods of 10 and 15 days, the ODM-oil group reveals a significantly higher ALP activity expression than ODFM group and ODM group. * *p* < 0.05, ** *p* < 0.01. (Note ODFM: odontogenesis induction-free medium; ODM: odontogenesis induction medium; and ODM-oil: odontogenesis induction medium with *S. mukorossi* seed oil.).

Figure 6 shows the measurement results obtained for the alkaline phosphatase activity of the ODFM, ODM and ODM-oil groups after culture periods of 5, 10 and 15 days. For all of the culture periods, the DPSCs cultured in ODM exhibit a higher ALP activity expression than those cultured in OFDM (*p* < 0.05). No significant difference is observed between the ODM group and ODM-oil group after 5 days. However, after ten days, the ALP activity of the ODM-oil group (0.533 ± 0.056 μM pi/mg protein/min) is significantly higher than that of the ODFM group (0.154 ± 0.021 μM pi/mg protein/min) and ODM group (0.242 ± 0.031 μM pi/mg protein/min) (*p* < 0.01). The same result is observed after the maximum incubation period of 15 days.

### 3.5. Real-Time PCR Assay

**Figure 7:**
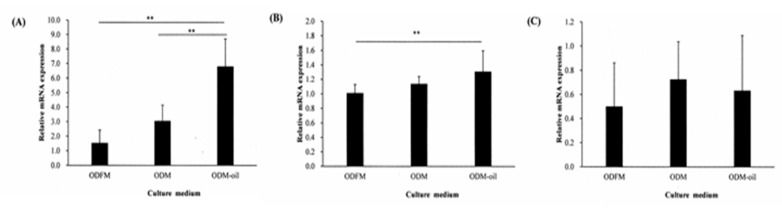
Expressions of odontogenic genes: (**A**) ALP, (**B**) BMP2 and (**C**) DMP-1 following culturing of DPSCs with ODFM, ODM and ODM-oil for 48 h. ** *p* < 0.01. (Note ODFM: odontogenesis induction-free medium; ODM: odontogenesis induction medium; and ODM-oil: odontogenesis induction medium with *S. mukorossi* seed oil.).

Figure 7 shows the effects of *S. mukorossi* seed oil addition on the expressions of osteogenic and odontogenic genes (ALP, BMP-2 and DMP-1) of the DPSCs cultured in different ODM media for 48 h. For the ALP gene (Figure 7A), the expression of the DPSCs cultured in ODM-oil is significantly higher than that of the cells cultured in ODFM or ODM (*p* < 0.01). However, no significant difference is observed between the ODFM and ODM groups. For the BMP-2 gene (Figure 7B), the expression of the DPSCs cultured in ODM-oil is significantly higher than that of the cells cultured in ODFM (*p* < 0.05) but is not significantly different from that of the cells cultured in ODM. For the DMP-1 gene (Figure 7C), no significant difference is observed among the three experimental groups.

### 3.6. Scanning Electron Microscopy Imaging 

**Figure 8:**
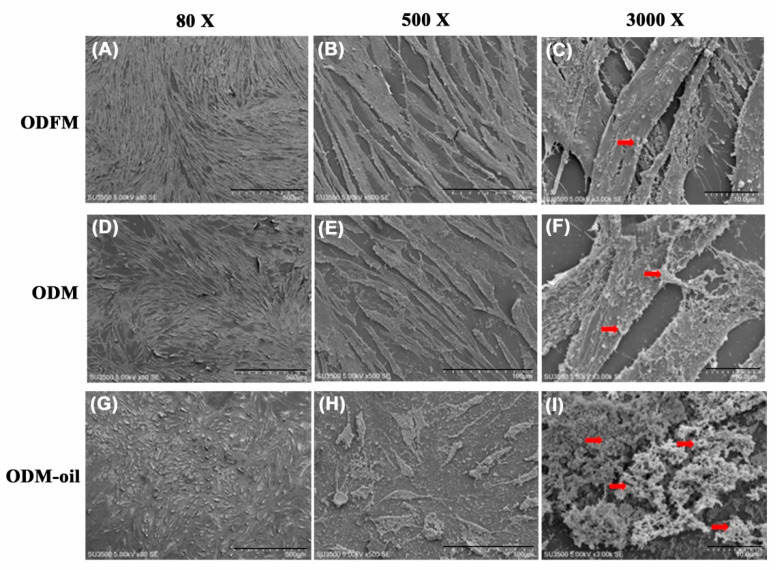
Scanning electron microscopy images of DPSCs cultured in ODFM (**A**–**C**), ODM (**D**–**F**) and ODM-oil (**G**–**I**) for 7 days. The DPSCs cultured with odontogenic medium and *S. mukorossi* seed oil secrete more abundant extracellular granules and vesicles (red arrows). (Note ODFM: odontogenesis induction-free medium; ODM: odontogenesis induction medium; and ODM-oil: odontogenesis induction medium with *S. mukorossi* seed oil.). Scale bars demote 500, 100 and 10 µm for magnifications of 80× (**A**, **D** and **G**), 500× (**B**, **E** and **H**) and 3000× (**C**, **F** and **I**).

Figure 8 presents SEM images of the DPSC morphology, attachment and structure after 14 days of culturing in ODFM, ODM and ODM-oil. As shown, the DPSCs cultured in ODFM have a spindle-like and fibroblastic appearance (Figure 8A–C). For the cells cultured in ODM, the DPSCs maintain a spindle-like shape (Figure 8D,E). However, matrix vesicles released from the cells are also observed (Figure 8F). Finally, following culturing in ODM-oil, the DPSCs have a more extended and mature morphology (Figure 8G) with greater filopodia formed around the cell periphery (Figure 8H). In addition, the cells change from a polygonal shape to a more stellar shape (Figure 8H). Furthermore, abundant extracellular granules and vesicle depositions are released from the cells (Figure 8I) and accumulate on and around the cells (Figure 8H).

## 4. Discussion

The application of plant-derived compounds and seed oils to the field of stem cell therapy and tissue engineering has attracted significant attention in recent years [14,32]. Previous studies on *S. mukorossi* have focused mainly on the biological properties of saponins; in particular, their tumor growth inhibiting effects and anti-oxidative, anti-cardiovascular and anti-inflammatory properties [19,27,30]. By contrast, the effects of *S. mukorossi* seed oil on the proliferation and differentiation of DPSCs have attracted scant attention. Accordingly, this study has performed a systematic investigation into the effects of *S. mukorossi* seed oil on the proliferation, osteogenic/odontogenic differentiation and matrix vesicle secretion properties of DPSCs.

DPSCs are distinguished by their ability to differentiate into both osteoblasts and odontoblasts, and thus play a critical role in maintaining bone/dentin remodeling. However, this ability is easily impaired due to increase in oxidative stress or inflammation. It has been reported that production of extracellular calcium deposits in vitro is a signal of osteogenesis. The present study has shown that *S. mukorossi* seed oil markedly improves the mineralization capacity of DPSCs after 10 days of induction with osteogenic or odontogenic medium (Figure 4 and Figure 5). This osteogenesis-promoting effect of *S. mukorossi* is consistent with the results reported for other natural plant-derived compounds in previous studies [33,34,35]. 

Cell-derived extracellular particles, generally referred to as matrix vesicles (MVs), can mediate cell-to-cell communication and are associated with physiological mineralization in bone, cartilage and dentin [36,37] due to the role they play in acting as nucleation sites for the formation of hydroxyapatite crystals [37]. The present results have shown that the secretion of extracellular nodules from DPSCs is enhanced following 14 days of ODM-oil induction (Figure 8H). Furthermore, since MVs contain abundant ALP [38], the DPSCs treated with *S. mukorossi* seed oil have shown a high ALP activity (Figure 6). ALP is an early marker for the differentiation of bone precursor cells, and hence a higher ALP activity indicates an improved differentiation capacity [39]. Notably, the present results have shown that *S. mukorossi* seed oil not only increases the ALP activity of the DPSCs (Figure 6), but also its gene expression (Figure 7A). Combined with the SEM evidence that DPSCs treated with *S. mukorossi* seed oil exhibit a more mature morphology (Figure 8H,I) and release more calcium deposits (Figure 4 and Figure 5), it is reasonable to infer that *S. mukorossi* seed oil has a positive effect on both the osteogenic/odontogenic differentiation capacity of DPSC and the matrix vesicle secretion of **odontogenic** medium-treated DPSCs. 

Bone morphogenetic proteins (BMPs) is an important cytokine involved tooth development. It was reported that increased BMP-2 expression is a sign during the differentiation of odontoblasts [40]. It can induce the expression of odontoblastic differentiation markers after implantation into dental papilla in vitro and induces reparative dentin on amputated pulp in vivo [40]. However, although ALP, BMP-2 and DMP-1 are cytokines for stimulating osteoblast/odontoblast cell differentiation [33], the present results have shown that *S. mukorossi* seed oil enhances bone/dentin mineralization mainly by increasing the ALP gene expression (Figure 7A) and enzyme activity (Figure 6). That is, it has no obvious effect on the expression of BMP-2 (Figure 7B) or DMP-1 (Figure 7C). The maturation of odontoblast can be divided into two stages, namely stem cell-to-preodontoblast and preodontoblast-to mature odontoblast. During both stages, BMP-2 serves as a regulator for differentiating the DPSCs into odontoblastic lineage and stimulating the formation of reparative dentin [41]. Furthermore, during the pulp healing process, pulp cells migration and BMPs released from the surrounding dentin are two key steps [42]. That is, the DPSCs are the receptors of BMP-2, rather than the major donor. This then accounts for the more minor BMP-2 gene expression of the DPSCs treated with ODM-oil than those treated with only ODM in the present study (Figure 7B). In particular, the culture period of the current experiment is insufficiently long to allow the stem cells to mature into odontoblasts. Unlike BMP-2 and DMP-1, which are mainly released by mature odontoblasts, the formation of MVs can be found not only in osteoblasts, but also in pre-odontoblast-initiated mineralization [43]. These results therefore suggest that *S. mukorossi* seed oil mediates bone formation and dentin generation only in the early maturation stage.

The present results have shown that *S. mukorossi* seed oil has little effect on the proliferation of DPSCs (Figure 3A,B). When the seeding density was 2 × 10^4^ cells/mL, the detected OD values significantly increase to about 0.4 at day 5 (Figure 3A). However, the cell viability reached this value at day 3 when the seeding density increased to 5 × 10^4^ cells/mL. For both culture densities, no significant difference in the cell viabilities of the two groups is observed over the entire 7-day incubation period. This finding is very different from that of a previous study, which concluded that *S. mukorossi* seed oil promotes the growth of skin cells and subsequent wound healing [19]. The apparent discrepancy between the two studies may stem from the fact that the previous work considered mature skin cells. Many studies have shown that the proliferation and differentiation of osteoprogenitor cells cannot be increased at the same time [39,44]. This most likely accounts for the present finding that the addition of *S. mukorossi* seed oil increases the osteogenesis and odontogenesis differentiation degree of DPSCs, but has no effect on their proliferation. 

It should be noted that the major molecular compound in *S. mukorossi* oil responsible for triggering the differentiation process in the DPSCs has not been identified in the present study. Mahmoudi et al. [20] investigated the proliferation and differentiation effects of *Alyssum homolocarpum* seed oil on stem cells, and concluded that β-sitosterol plays an important role in promoting the proliferation and differentiation of neural stem cells in vitro. β-sitosterol has also been reported to provide pharmacological and biological activities for several diseases without any undesirable side effects [45]. Interestingly, the phytochemical characteristics of the *S. mukorossi* seed oil used in the present study suggest the presence of abundant β-sitosterol [19]. However, this inference should be explored further in a future study.

## 5. Conclusions

In conclusion, this study has demonstrated that *S. mukorossi* seed oil enhances the osteogenic/odontogenic differentiation capacity of DPSCs by increasing their ALP gene expression, ALP activity and mineralization-related extracellular vesicle secretion. The improved stem cell differentiation potential induced by *S. mukorossi* seed oil is expected to be of benefit in furthering the use of DPSCs in performing regenerative repair in clinical applications. *S. mukorossi* seed oil can be an alternative reagent for the vital pulp therapy to stimulate the reparative dentin formation or combination with stem cell implantation for hard tissue engineering. Therefore, an in vivo study should go on to reveal the significant improvement of *S. mukorossi* to regenerative dentistry. 

## Figures and Tables

**Table 1:** Primers used for RT-PCR analysis.

Gene	Type	Primers	Accession	Product Length
ALP	Forward	5′-TAAGGACATCGCCTACCAGCTC-3′	XM_017000903.1	170
	Reverse	5′-TCTTCCAGGTGTCAACGAGGT-3′		
BMP2	Forward	5′- GAGAAGGAGGAGGCAAAGAAA-3′	NM_001200.4	181
	Reverse	5′- AGCAGCAACGCTAGAAGACAG-3′		
DMP-1	Forward	5′-ATGCCTATCACAACAAACC-3′	NM_001079911.3	100
	Reverse	5′-CTCCTTTATGTGACAACTGC-3′		
GAPDH	Forward	5′-GCACCGTCAAGGCTGAGAAC-3′	NM_001256799.3	138
	Reverse	5′-TGGTGAAGACGCCAGTGGA-3′		
